# Fire in the belly: A scoping review of the immunopathological mechanisms of acute pancreatitis

**DOI:** 10.3389/fimmu.2022.1077414

**Published:** 2023-01-11

**Authors:** Karthik Venkatesh, Hannah Glenn, Anthony Delaney, Christopher R. Andersen, Sarah C. Sasson

**Affiliations:** ^1^Malcolm Fisher Department of Intensive Care, Royal North Shore Hospital, St Leonards, NSW, Australia; ^2^The Kirby Institute, The University of New South Wales, Kensington, NSW, Australia; ^3^Division of Critical Care, The George Institute for Global Health, Newtown, NSW, Australia; ^4^Institute of Clinical Pathology and Medical Research, Westmead Hospital, Westmead, NSW, Australia

**Keywords:** acute pancreatitis (AP), immunopathology, human, immune dysregulation, scoping review, immunodeficiency

## Abstract

**Introduction:**

Acute pancreatitis (AP) is characterised by an inflammatory response that in its most severe form can cause a systemic dysregulated immune response and progression to acute multi-organ dysfunction. The pathobiology of the disease is unclear and as a result no targeted, disease-modifying therapies exist. We performed a scoping review of data pertaining to the human immunology of AP to summarise the current field and to identify future research opportunities.

**Methods:**

A scoping review of all clinical studies of AP immunology was performed across multiple databases. Studies were included if they were human studies of AP with an immunological outcome or intervention.

**Results:**

205 studies met the inclusion criteria for the review. Severe AP is characterised by significant immune dysregulation compared to the milder form of the disease. Broadly, this immune dysfunction was categorised into: innate immune responses (including profound release of damage-associated molecular patterns and heightened activity of pattern recognition receptors), cytokine profile dysregulation (particularly IL-1, 6, 10 and TNF-α), lymphocyte abnormalities, paradoxical immunosuppression (including HLA-DR suppression and increased co-inhibitory molecule expression), and failure of the intestinal barrier function. Studies including interventions were also included. Several limitations in the existing literature have been identified; consolidation and consistency across studies is required if progress is to be made in our understanding of this disease.

**Conclusions:**

AP, particularly the more severe spectrum of the disease, is characterised by a multifaceted immune response that drives tissue injury and contributes to the associated morbidity and mortality. Significant work is required to develop our understanding of the immunopathology of this disease if disease-modifying therapies are to be established.

## Introduction

Acute pancreatitis (AP) is an inflammatory syndrome of the pancreas with a spectrum that ranges from mild, self-limiting disease to severe illness characterised by systemic inflammation and persistent multi-organ failure. It has a highly variable incidence of 3-130 per 100,000 people, with a rising incidence observed in developed countries (1). While the majority (80%) of cases are mild and typically resolve over a few days, the remaining 20% develop severe acute pancreatitis (SAP), which is defined by the development of organ failure (single or multiple) that persists for greater than 48 hours (**Table 1**) (2). In addition to local complications such as acute pancreatic fluid collections and pseudocyst formation (3), SAP is associated with peripancreatic vascular complications (e.g. splanchnic venous thrombosis, pseudo-aneurysm), abdominal compartment syndrome, acute respiratory distress syndrome (ARDS), and acute kidney injury (4). In the acute phase, SAP typically requires intensive care unit (ICU) admission for ventilatory support, vasopressor therapies or renal replacement therapy. A subset of SAP patients develops pancreatic necrosis, which may become infected and predispose to sepsis. The early mortality in SAP arises from the acute inflammatory response and organ dysfunction, while delayed mortality is often due to sepsis from infected necroses with an associated mortality rate of 20-30% despite optimal care (5, 6).

**Table 1:** Pancreatitis Severity Classification based on the Revised Atlanta 2012 Consensus criteria (2).

Pancreatitis Severity Grade
**Mild acute pancreatitis (MAP)** No organ failure No local or systemic complications
**Moderately severe acute pancreatitis (MSAP)** Organ failure that resolves within 48 hours (transient organ failure) AND/OR Local or systemic complications without persistent organ failure
**Severe acute pancreatitis (SAP)** Persistent organ failure (> 48 hour) Single organ failure Multiple organ failure

The initial pancreatic injury is multifactorial including enzyme-induced autodigestion, and derangements in intracellular metabolic pathways including impaired calcium signalling, mitochondrial dysfunction and impaired autophagy (7); all of which may trigger tissue necrosis. Why there is a subsequent dichotomy between the development of mild and severe disease is not well understood, but there is evidence that SAP is contributed to by dysregulated inflammatory and immunological processes in response to the initial tissue injury. Despite significant efforts in animal and human studies to better understand disease pathogenesis, the mechanisms are still not well understood and as such there are no specific disease-modifying and/or targeted therapies. While animal models have been valuable in developing an understanding of AP, there are limitations in the translation of these findings into clinical practice (8). Further, the heterogeneity of the AP patient population and the variable timing of hospital presentation limit the ability to easily conduct clinical trials of potential therapies.

Understanding the cellular and molecular mechanisms driving inflammation in early SAP could provide opportunities for targeted, host-directed therapies. Studies to-date suggest that the pathways are multifactorial and include genetic predisposition, abnormal innate immune and cellular responses, paradoxical immunosuppression, and alterations to gut permeability causing microbial translocation, as detailed below. To progress our understanding of the immunological and inflammatory mechanisms driving SAP, further robust pre-clinical human translational studies are required. The purpose of this scoping review is to comprehensively investigate and summarise the data pertaining to immunopathological mechanisms in AP in adult humans, with a particular emphasis on distinguishing the mechanisms between SAP and the milder forms of the disease.

## Methods

A scoping review of the immunological mechanisms of acute pancreatitis was performed to provide a narrative overview of the data rather than addressing a single, specific question. The protocol was drafted in conjunction with the Preferred Reporting Items for Systematic Reviews and Meta-analysis Protocols for Scoping Reviews (PRISMA-P ScR). The final protocol was registered with the Open Science Framework on 12 September 2021 (DOI 10.17605/OSF.IO/UEYNS).

### Database search

A comprehensive search strategy was developed to investigate acute pancreatitis in adult humans, using MeSH terms for a number of immunological mechanisms (see **Supplementary Data A**). A search was conducted through Medline (PubMed), Web of Science, Cochrane Registry, and the National Institutes of Health Clinical Trials Registry. The time frame for the search was limited to January 1980 to May 2021 given the technical and historical limitations in investigating immunological mechanisms prior to this. For practical purposes only studies published in English were considered for inclusion.

Studies were included if they investigated an immunological mechanism, intervention or outcome relating to acute pancreatitis in humans. Animal studies, paediatric studies and studies of chronic pancreatitis were excluded. We also excluded studies investigating auto-immune pancreatitis due to there being a distinct immunological mechanism and treatment for that syndrome. Review articles, meta-analyses and case reports were excluded however their reference lists were reviewed to ensure relevant studies were not omitted.

### Publication selection

Studies were imported into Covidence^©^ (Melbourne, Australia) review management software and duplicates were removed. Title and abstract screening was performed by two authors independently. Full text screening was subsequently performed by the two authors and reference lists for relevant publications were also screened for study inclusion. Any uncertainty or conflict regarding study inclusion was then deferred to a third author for consensus. Data was extracted and collected in Covidence^©^ (see data extraction form in **Supplementary Data B**) using a pre-specified data dictionary.

### Data synthesis

Simple quantitative analysis was performed to measure trial demographics and patient characteristics, including median values, percentages and interquartile ranges. We also quantified disease factors including symptom duration prior to hospital presentation, method of classifying disease severity and aetiology of AP. Studies were analysed to quantify timing and duration of outcome measurement and qualitative analysis was performed on immunological assays and outcomes.

## Results

### Search results

**Figure 1:**
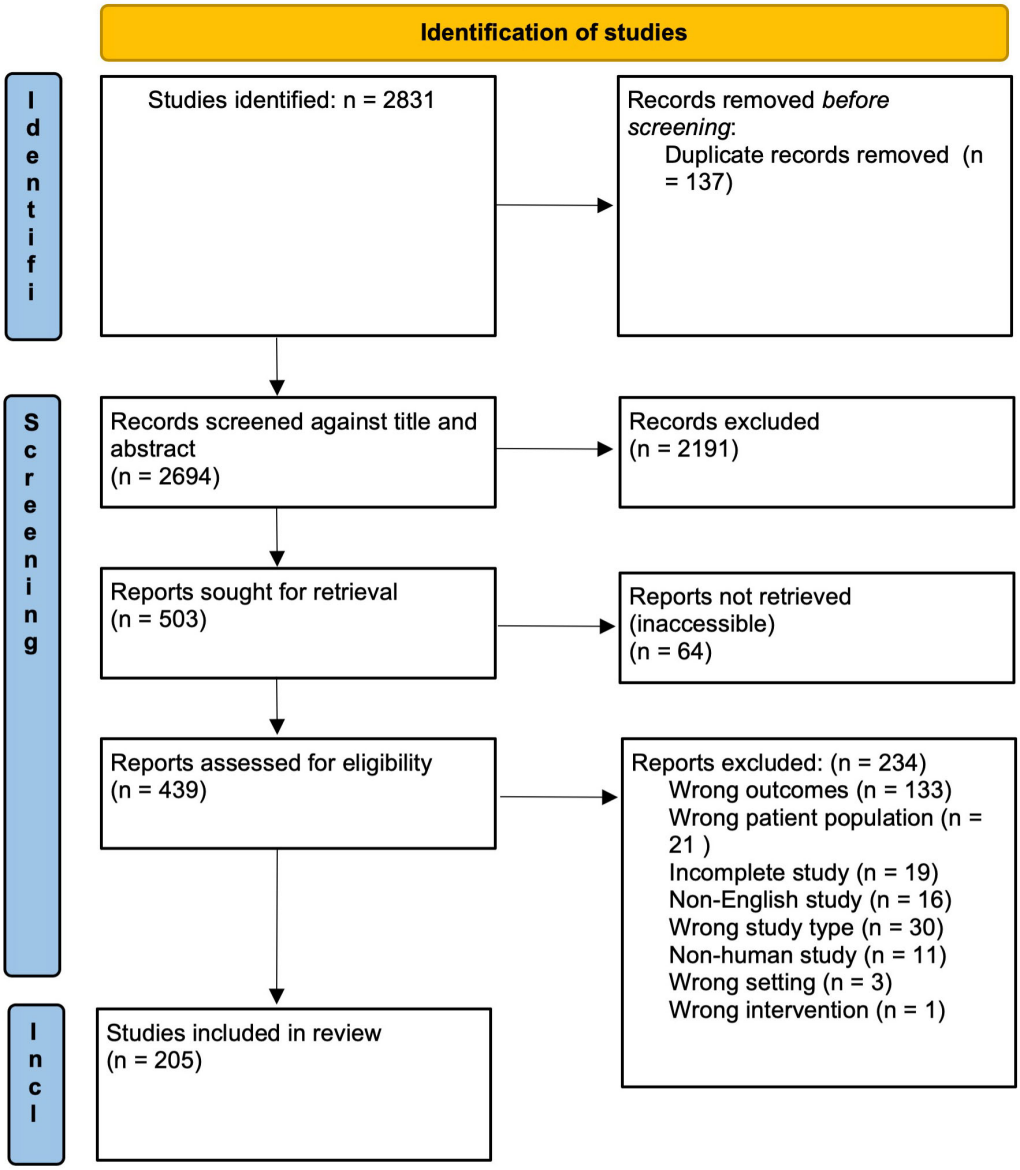
PRISMA flow diagram of study screening and selection. Adapted from The PRISMA 2020 statement: an updated guideline for reporting systematic reviews (9).

A total of 2831 studies were identified after the initial search. After removal of 137 duplicates, 2489 studies were removed during abstract screening and full text review, with 205 studies remaining for data analysis (see PRISMA flow diagram – **Figure 1**).

### Study characteristics

Of the 205 retrieved studies (see **Table 2**), 17 (8.3%) were randomised controlled trials (RCTs), 9 (4.4%) pseudo-randomised controlled trials, 9 (4.4%) randomised non-controlled intervention study, 1 (0.5%) retrospective review of RCT data, 99 (48.3%) case-control studies, 68 (33.2%) case series, and 2 (1%) cohort studies. There were a total of 23,206 participants (including controls) across the 205 studies. The time frame for retrieved studies was 1984 to 2020. The majority of studies, 190 (93%), were conducted in a single centre. The median number of participants per trial was 62 (IQR 40-102). 129 (63%) of the studies had a control cohort. Of the 205 studies, 77 (38%) did not specify a time frame for symptom onset for inclusion.

**Table 2:** All studies identified in initial search by primary focus area - innate immunity, cytokines, genetics, leukocyte-mediated, cytokine, gut permeability, or intervention.

Author	Year	Country	Study design	Population severity	Control
Innate immunity					
Arriaga-Pizano (10)	2018	Mexico	Case series	Mild; Moderate; Severe	No
Chen (11)	2017	China	Case control study	Mild; Moderate; Severe	Yes
Dabrowski (12)	2014	Poland	Case control study	Mild; Severe	Yes
Ferat-Osorio (13)	2009	Mexico	Case control study	Mild; Severe	Yes
Gong (14)	2017	China	Case control study	Mild; Severe	Yes
Götzinger (15)	2000	Austria	Case control study	Mild; Severe	Yes
Ho (16)	2006	Taiwan	Case series	Severe	No
Huang (17)	2015	China	Case control study	Mild; Moderate; Severe	Yes
Kaufmann (18)	1996	Austria	Case series	Severe	Yes
Kocsis (19)	2009	Hungary	Case control study	Mild; Severe	Yes
Kylänpää (20)	2005	Finland	Case series	Severe	Yes
Kylänpää-Bäck (21)	2001	Finland	Case control study	Mild; Moderate; Severe	Yes
Li (22)	2007	China	Case control study	Mild; Severe	Yes
Li (23)	2012	China	Case control study	Severe	Yes
Lindstrom (24)	2006	Finland	Case control study	Moderate; Severe	Yes
Liu (25)	2017	United Kingdom	Case control study	Mild; Moderate; Severe	Yes
Maksimow (26)	2014	Finland	Case control study	Mild; Moderate; Severe	Yes
McMahon (24)	1984	England	Case control study	Mild; Severe	Yes
Nakae (27)	2001	Japan	Case series	Mild; Moderate; Severe	No
Pan (28)	2017	China	Case control study	Mild; Moderate; Severe	Yes
Pérez (29)	2019	Spain	Case control study	Mild; Moderate	Yes
Rahman (30)	2007	England	Case control study	Mild; Severe	Yes
Rau (31)	2003	Germany	Case series	Mild; Severe	No
Richter (32)	1999	Germany	Case series	Mild; Severe	No
Satoh (33)	2003	Japan	Case series	Mild; Severe	Yes
Satoh (34)	2002	Japan	Case series	Mild; Severe	No
Shao (35)	2012	Germany	Case control study	Mild; Moderate; Severe	Yes
Shinzeki (36)	2007	Japan	Case series	Severe	No
Szatmary (37)	2017	England	Case series	Mild; Moderate; Severe	No
Vlachos (38)	2014	Greece	Case control study	Mild; Severe	Yes
Yasuda (39)	2008	Japan	Case control study	Not specified	Yes
Yu (40)	2004	China	Case series	Mild; Moderate; Severe	No
Zhang (41)	2018	China	Case control study	Mild; Severe	Yes
Zhang (42)	2019	China	Case control study	Mild; Moderate; Severe	Yes
Cytokine					
Andersson (43)	2010	Sweden	Case series	Mild; Severe	No
Banks (2)	1991	United Kingdom	Case control study	Mild; Severe	Yes
Berney (44)	1999	Switzerland	Case series	Mild; Severe	No
Brivet (45)	1999	France	Case series	Mild; Severe	No
Cíeranicí (46)	2020	Slovenia	Case series	Mild; Severe	No
Chen (47)	1999	Taiwan	Case series	Mild; Severe	No
Chen (48)	2006	China	Case control study	Mild; Severe	Yes
Dambrauskas (49)	2010	Lithuania	Case control study	Mild; Severe	Yes
Daniel (50)	2010	Poland	Case control study	Severe	Yes
de Beaux (51)	1996	Scotland	Case series	Mild; Severe	No
de Beaux (52)	1996	Scotland	Case control study	Mild; Severe	Yes
Duarte-Rojo (53)	2009	Mexico	Case series	Mild; Severe	No
Gunjaca (54)	2012	Croatia	Case series	Mild; Severe	No
Heath (55)	1993	Scotland	Case series	Mild; Severe	No
Heath (56)	1995	Scotland	Case series	Mild; Severe	No
Hirota (57)	2000	Japan	Case series	Mild; Severe	No
Ho (58)	2011	Taiwan	Case control study	Mild; Severe	Yes
Hynninen (59)	2000	Finland	Case series	Severe	No
Ikei (60)	1998	Japan	Case series	Mild; Moderate; Severe	No
Inagaki (61)	1997	Japan	Case series	Mild; Moderate; Severe	No
Jamdar (62)	2006	England	Case control study	Severe	Yes
Jia (63)	2015	China	Case control study	Mild; Moderate; Severe	Yes
Jiang (64)	2004	Taiwan	Case series	Mild; Severe	No
Karrasch (65)	2015	Germany	Case control study	Mild; Severe	Yes
Kaufmann (66)	1997	Austria	Case series	Mild; Severe	No
Kaw (67)	2001	USA	Case series	Mild; Moderate; Severe	No
Kolber (68)	2018	Poland	Case series	Mild; Moderate; Severe	No
Kylänpää-Bäck (69)	2001	Finland	Case series	Mild; Severe	No
Laveda (70)	2005	Spain	Case control study	Mild; Severe	Yes
Lin (71)	2019	Turkey	Case control study	Mild; Severe	Yes
Malmstrom (72)	2012	Denmark	Case series	Mild; Severe	No
Mayer (73)	2000	Germany	Case series	Mild; Severe	No
McKay (74)	1996	Scotland	Case series	Moderate; Severe	No
Mentula (75)	2004	Finland	Case series	Mild; Moderate; Severe	No
Mentula (76)	2005	Finland	Case control study	Moderate; Severe	Yes
Messmann (77)	1997	Germany	Cohort study	Mild; Severe	No
Messmann (78)	1998	Germany	Case control study	Mild; Severe	Yes
Simovic (79)	1999	New Zealand	Case series	Mild; Severe	No
Montravers (80)	1995	France	Case series	Severe	No
Nakae (81)	2003	Japan	Case series	Mild; Moderate; Severe	No
Naskalski (82)	2003	Poland	Case series	Mild; Severe	No
Nieminen (83)	2014	Finland	Case series	Mild; Moderate; Severe	No
O’Reilly (84)	2006	England	Case control study	Mild; Severe	Yes
Oezcueruemez-Porsch (85)	1998	Germany	Case series	Not specified	No
Paajanen (86)	1995	Finland	Case control study	Mild; Severe	Yes
Panek (87)	2006	Poland	Case series	Mild; Severe	No
Papachristou (88)	2006	USA	Case series	Mild; Severe	No
Park (89)	2015	Korea	Case series	Mild; Moderate	No
Peng (90)	2015	Taiwan	Case series	Severe	No
Pezzilli (91)	1995	Italy	Case series	Mild; Severe	Yes
Pezzilli (92)	1997	Italy	Case control study	Mild; Severe	Yes
Pezzilli (93)	1999	Italy	Case control study	Mild; Severe	Yes
Pezzilli (94)	1999	Italy	Case control study	Mild; Severe	Yes
Pongratz (95)	2013	Germany	Case series	Mild; Severe	No
Pooran (96)	2003	USA	Case control study	Mild; Severe	Yes
Rau (97)	2001	Germany	Case series	Mild; Severe	No
Regnér (98)	2008	Sweden	Case series	Mild; Severe	No
Rodriguez-Nicolas (99)	2018	Spain	Case control study	Mild; Moderate; Severe	Yes
Sathyanarayan (100)	2006	India	Case series	Mild; Severe	Yes
Sempere (101)	2008	Spain	Case control study	Mild; Severe	Yes
Shen (102)	2015	China	Case series	Severe	No
Shokuhi (103)	2002	England	Case control study	Mild; Severe	Yes
Sternby (104)	2016	Sweden	Case series	Mild; Severe	No
Stimac (105)	2006	Croatia	Case series	Mild; Severe	No
Thomson (106)	2019	South Africa	Case control study	Mild; Moderate; Severe	Yes
Ueda (107)	2006	Japan	Case control study	Moderate; Severe	Yes
Ueda (108)	2007	Japan	Case control study	Mild; Severe	Yes
Uehara (109)	2003	Japan	Case control study	Mild; Severe	Yes
Vasseur (110)	2014	France	Case series	Mild; Severe	No
Viedma (111)	1992	Spain	Case series	Mild; Severe	No
Wereszczynska-Siemiatkowska (112)	2002	Poland	Case control study	Mild; Severe	Yes
Wereszczynska-Siemiatkowska (113)	2003	Poland	Case control study	Mild; Severe	Yes
Wereszczynska-Siemiatkowska (114)	2004	Poland	Case control study	Mild; Severe	Yes
Yang (115)	2015	China	Case series	Moderate; Severe	No
Zhang (116)	2018	China	Case control study	Mild; Moderate; Severe	Yes
Genetics					
Balog (117)	2005	Hungary	Case control study	Mild; Severe	Yes
Bao (118)	2015	China	Case control study		Yes
Bishehsari (119)	2012	USA	Case control study	Mild; Severe	Yes
Bishu (120)	2018	USA	Case control study	Mild; Moderate; Severe	Yes
de-Madaria (121)	2008	Spain	Case series	Mild; Severe	No
Liu (122)	2014	China	Case control study	Mild; Severe	Yes
Makhija (123)	2007	United Kingdom	Case control study	Mild; Moderate; Severe	Yes
Masamune (124)	2010	Japan	Case control study	Mild; Moderate; Severe	Yes
Matas-Cobos (125)	2015	Spain	Case control study	Mild; Moderate; Severe	Yes
Ozhan (126)	2010	Turkey	Case control study	Mild; Severe	Yes
Papachristou (127)	2005	USA	Case control study	Mild; Severe	Yes
Powell (128)	2001	Scotland	Case control study	Mild; Severe	Yes
Rodriguez-Nicolas (129)	2019	Spain	Case control study	Mild; Moderate; Severe	Yes
Sargen (130)	2000	England	Case control study	Mild; Severe	Yes
Smithies (131)	2000	England	Case control study	Mild; Severe	Yes
Takagi (132)	2009	Japan	Case control study	Mild; Severe	Yes
Tukiainen (133)	2008	Finland	Case control study	Mild; Severe	Yes
Zhang (134)	2008	China	Case control study	Mild; Severe	Yes
Leukocyte-mediated					
Algaba-Chueca (135)	2017	Spain	Case control study	Mild; Moderate; Severe	Yes
Bhatnagar (136)	2001	India	Case series	Severe	Yes
Bhatnagar (137)	2001	India	Case control study	Severe	Yes
Bidarkundi (138)	2002	India	Case control study	Mild; Severe	Yes
Chen (139)	2005	China	Case control study	Mild; Severe	Yes
Curley (140)	1996	United Kingdom	Case control study	Mild; Severe	Yes
Curley (141)	1993	United Kingdom	Case control study	Mild; Severe	Yes
Dabrowski (142)	2008	Poland	Case control study	Mild; Severe	Yes
Li (143)	2013	China	Case series	Severe	No
Minkov (144)	2017	Bulgaria	Case control study	Mild; Moderate; Severe	Yes
O’Neill (145)	2000	Ireland	Case control study	Mild; Severe	
Oiva (146)	2010	Finland	Case control study	Severe	Yes
Pezzilli (147)	2003	Italy	Case control study	Mild; Severe	Yes
Pezzilli (148)	1995	Italy	Case control study		Yes
Pezzilli (149)	1997	Italy	Case control study	Mild; Severe	Yes
Pietruczuk (150)	2006	Poland	Case control study	Mild; Severe	Yes
Salomone (151)	2002	Italy	Case control study	Mild; Moderate; Severe	Yes
Shen (152)	2015	China	Case series	Mild; Moderate; Severe	No
Susak (153)	2021	Ukraine	Case control study	Mild; Moderate; Severe	Yes
Sweeney (154)	2003	Ireland	Case control study	Mild	Yes
Takeyama (155)	2000	Japan	Cohort study	Severe	No
Ueda (156)	2006	Japan	Case series	Severe	No
Wang (157)	2017	China	Case series	Mild; Moderate; Severe	No
Widdison (158)	1996	England	Case control study	Mild; Severe	Yes
Zhang (159)	2014	China	Case series	Mild; Severe	No
Zhang (160)	2017	China	Case control study	Mild	Yes
Zhao (161)	2019	China	Case control study	Mild; Moderate; Severe	Yes
Gut permeability					
Ammori (162)	1999	United Kingdom	Case control study	Mild; Severe	Yes
Buttenschoen (163)	2000	Germany	Case control study	Mild; Severe	Yes
Kivilaakso (164)	1984	Finland	Case control study	Mild; Moderate; Severe	Yes
Liu (165)	2008	China	Case control study	Mild; Moderate; Severe	Yes
Penalva (166)	2004	Spain	Case control study	Mild; Severe	Yes
Rahman (167)	2003	England	Case control study	Mild; Severe	Yes
Tan (168)	2015	China	Case control study	Mild; Moderate	Yes
Windsor (169)	1993	Scotland	Case series	Mild; Severe	No
Intervention					
Abe (170)	2010	Japan	Retrospective case control study	Severe	Yes
Al-Leswas (171)	2020	England	Randomised controlled trial	Severe	Yes
Arutla (172)	2019	India	Randomised controlled trial	Mild; Moderate; Severe	Yes
Chen (173)	2019	China	Pseudo-RCT	Severe	No
Chen (174)	2016	China	Retrospective review of RCT data	Severe	Yes
Chooklin (175)	2009	Ukraine	Randomised controlled trial	Mild; Severe	Yes
Chu (176)	2013	China	Randomised non-controlled intervention study	Severe	No
Cui (177)	2014	China	Case series	Severe	No
Dai (178)	2015	China	Case control study	Moderate; Severe	Yes
Eckerwall (179)	2006	Switzlerand	Randomised controlled trial	Severe	Yes
Gao (180)	2018	China	Pseudo-RCT	Severe	Yes
Gorsky (181)	2015	Russia	Randomised controlled trial	Mild; Severe	Yes
Guo (182)	2020	China	Pseudo-RCT	Severe	Yes
Guo (183)	2014	China	Pseudo-RCT	Severe	Yes
Guo (184)	2016	China	Randomised controlled trial	Severe	Yes
Gupta (185)	2003	United Kingdom	Pseudo-RCT	Severe	No
He (186)	2013	China	Case series	Severe	No
Huang (187)	2019	China	Randomised non-controlled intervention study	Severe	No
Ino (188)	2008	Japan	Case series	Severe	No
Johnson (189)	2001	United Kingdom	Randomised controlled trial	Severe	Yes
Kingsnorth (190)	1995	United Kingdom	Randomised controlled trial	Mild; Severe	Yes
Kyhala (191)	2012	Finland	Randomised controlled trial	Severe	Yes
Li (192)	2020	China	Pseudo-RCT	Severe	Yes
Mora (193)	2007	Spain	Pseudo-RCT	Severe	No
Ockenga (194)	2002	Germany	Randomised controlled trial	Moderate; Severe	Yes
Oda (195)	2005	Japan	Case series	Severe	No
Powell (196)	2000	United Kingdom	Pseudo-RCT	Mild; Severe	Yes
Sharma (197)	2011	India	Randomised controlled trial	Not specified	Yes
Singh (198)	2014	India	Randomised controlled trial	Mild; Severe	Yes
Sun (199)	2013	China	Randomised non-controlled intervention study	Severe	No
Tang (200)	2007	China	Pseudo-RCT	Severe	Yes
Vege (201)	2015	USA	Randomised controlled trial	Severe	Yes
Wan (202)	2014	China	Randomised non-controlled intervention study	Severe	No
Wang (203)	2016	China	Randomised non-controlled intervention study	Severe	No
Wang (204)	2009	China	Randomised controlled trial	Severe	Yes
Wang (205)	2013	China	Randomised non-controlled intervention study	Severe	No
Wang (206)	2008	China	Randomised non-controlled intervention study	Severe	No
Wang (207)	2019	China	Randomised controlled trial	Severe	Yes
Wang (208)	2017	China	Retrospective case control study	Severe	Yes
Windsor (209)	1998	England	Randomised non-controlled intervention study	Mild; Moderate; Severe	No
Yang (210)	2010	China	Randomised controlled trial	Severe	Yes
Zhao (211)	2003	China	Randomised non-controlled intervention study	Severe	No
Zhao (212)	2013	China	Randomised controlled trial	Severe	No

**Figure 2:**
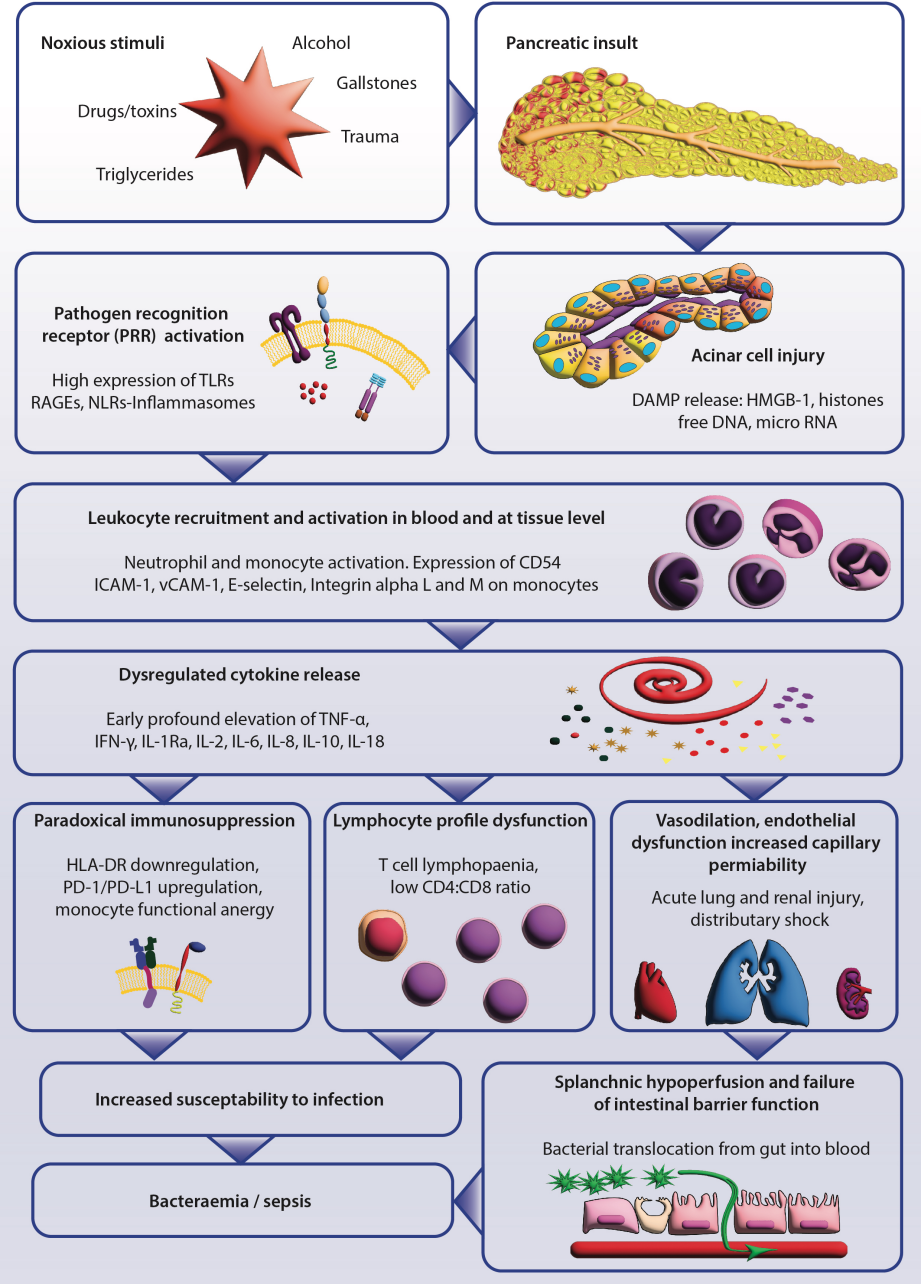
Summary of the current understanding of the immunopathogenesis of severe acute pancreatitis (SAP). The initial pancreatic injury can be instigated by a variety of noxious stimuli; acinar cell injury releases damage-associated molecular patterns (DAMPs). DAMPs are recognised by pattern-recognition receptors (PRR), which activate tissue and peripheral blood leukocytes and triggers the inflammatory cytokine release. The sequelae include alterations in lymphocyte phenotype, paradoxical hallmarks of immunosuppression, and a systemic inflammatory syndrome of endothelial dysfunction. Increased vascular permeability that causes distributory shock and multi-organ failure also occurs. Local and systemic factors induce failure of the intestinal barrier, promoting bacterial translocation. The relative immunodeficiency and increased intestinal permeability significantly increase the risk of secondary infectious complications.

During the full text screening process, six recurring themes emerged and provided a structure for the review. These themes were: aberrant innate immune response, cytokine profile, cellular responses, genetic predisposition, alterations to intestinal barrier function, and intervention studies. The main immunopathological findings are summarised in a flow chart in **Figure 2**.

### Study demographics

The studies included were conducted across multiple countries and regions – of note 24% occurred in China, 9% in Japan, and 8% in Finland (**Table 3**). While these countries do have relatively higher incidences of AP, these figures do not reflect the global distribution of the disease burden (1).

**Table 3:** Included studies by continent with number and percentages.

ASIA (n = 82, 40%)			EUROPE (N = 106, 51.7%)							
Country	Number	%	Country	Number		%	Country		Number	%
China	50	24.4%	Austria	3		1.5%	Ireland		2	1.0%
India	7	3.4%	Bulgaria	1		0.5%	Italy		8	3.9%
Japan	19	9.3%	Croatia	2		1.0%	Lithuania		1	0.5%
Korea	1	0.5%	Denmark	1		0.5%	Poland		9	4.4%
Taiwan	5	2.4%	England	12		5.9%	Russia		1	0.5%
OCEANIA (n = 1, 0.5%)			Finland	14		6.8%	Scotland		7	3.4%
Country	Studies	%	France	3		6.8	Slovenia		1	0.5%
New Zealand	1	0.5%	Germany	12		5.9%	Spain		11	5.4%
AMERICAS (n = 10, 4.9%)			Greece	1		0.5%	Switzerland		1	0.5%
Country	Number	%	Hungary	2		1.0%	UK		9	4.4%
Mexico	3	1.5%	AFRICA (n = 1, 0.5%)							
USA	7	3.4%	Country		Number			%		
			South Africa		1			0.5%		

Pancreatitis severity was classified using the Atlanta 2012 Consensus criteria in 110 (54%) of the studies). Most studies included a spectrum of disease: 97 (47%) mild and severe, 38 (18.5%) mild, moderate and severe, 6 (3%) moderate and severe, while 50 (24%) included severe disease only. With regards to aetiology, 33 studies (16%) did not specify an identified cause of AP in their patient population. Of the remaining studies that did specify, 166 studies (81%) specified gallstone/biliary aetiology, 164 (80%) alcohol, 59 (29%) hypertriglyceridaemia, 16 (8%) drug-induced, and 149 (70%) ‘other’ (which may have included less common pathologies for that particular population e.g. hypertriglyceridaemia or drugs that were specified in other study designs).

## Innate immune and inflammatory responses in AP

The initial pancreatic tissue injury triggers innate immune responses that promote the subsequent inflammatory cascade associated with AP. Of the included studies, 53 reported outcomes related to changes in the innate immune system in AP. Patients who develop SAP have profound derangements in these pathways compared to mild AP (MAP). These responses can be broadly categorised by the interaction of damage- associated molecular patterns (DAMPs) with pattern-recognition receptors (PRRs), early markers of granulocyte activation and adhesion as well as early markers of immunosuppression.

### Damage-associated molecular patterns

One of the evolved responses of the innate immune system is to recognise not only foreign pathogens but also to detect and respond to damage signals that may be indicative of host tissue injury. In AP, the initial tissue injury triggers the release of DAMPs into the blood, which typically occurs early in the acute phase of the illness. DAMPs directly interact with pattern-recognition receptors (PRRs), which are predominantly expressed by innate immune cells. These interactions trigger leukocyte activation and cytokine release, amplifying the inflammatory response to tissue injury but incidentally may also cause further tissue and organ dysfunction (213). Early DAMP release into the blood and their association with disease severity suggests that they may be useful biomarkers for prediction of severity on admission.

High-mobility group box-1 protein (HMGB-1) is a highly-conserved intranuclear protein normally involved in gene transcription but is also released into the circulation following cellular necrosis. Elevated HMGB-1 levels have been demonstrated in several inflammatory syndromes including sepsis, acute myocardial infarction and rheumatoid arthritis, and in this context is postulated to function as a pro-inflammatory mediator (152). In a case series of 15 patients with AP, HMGB-1 levels were significantly higher in patients with SAP compared to MAP, as well as in non-survivors compared with survivors (10). These elevations were observed from the time of admission and in the SAP cohort were sustained up to day 7 of illness. Similar findings were observed in a case-control study comparing admission levels of HMGB-1 in patients with MAP or SAP with a sepsis group and healthy controls (19). Both MAP and SAP had significantly higher HMGB-1 levels than healthy controls. In the SAP subgroup, HMGB-1 levels were elevated compared to the MAP patients, and were higher than levels expressed in the sepsis cohort. The early elevation of HMGB-1 and its correlation with disease severity suggests that it could be a useful biomarker for prediction of disease severity, and this requires further validation in larger studies.

Histones are intranuclear proteins involved in DNA packaging and gene regulation. In health, plasma histone levels are present at low levels, however elevated levels are seen in cellular injury syndromes such as trauma and sepsis (214). Elevated circulating histone levels have been implicated in early SAP. In a case-control study of 236 AP patients with 47 controls, circulating histone levels were comparable between volunteers, and patients with MAP or moderately severe AP (MSAP), however histone levels were significantly raised in the SAP cohort (25). In patients who presented with early organ failure (within 24 hours), histone levels were elevated compared to those with delayed organ failure. Similar findings were observed in another series where admission circulating histone levels were higher in SAP compared to MSAP and MAP (37). In both studies, receiver operating characteristics (ROC) area under the curve (AUC) for the accuracy of histones in predicting AP severity on admission were high (0.96 and 0.87 respectively). This suggests that plasma histones may be a useful biomarker of disease severity in AP, warranting further investigation.

Neutrophil extracellular traps (NETs) are expressed on neutrophil surfaces in response to stimulation by cytokine, lipopolysaccharide or intrinsic DAMPs. NETs are composed of decondensed DNA complexes and histones; their intrinsic purpose is an antimicrobial effect *via* the release of enzymes and proteins (a process known as NETosis). There is increasing evidence for the role of NETs in autoimmune and inflammatory disorders *via* direct tissue injury as well their potentiation of microvascular thrombosis and organ ischaemia (215). In AP, DAMP-induced NET activation appears to play a key role in both pancreatic and extra-pancreatic organ dysfunction. There is limited human data investigating NETs in AP, however one study assessed serum levels of free DNA and MPO-DNA complexes (markers of NET activity) in patients with SAP, MAP and healthy controls (216). The SAP cohort had significantly higher levels of both markers, which supports the implication of NETs in AP.

Other intranuclear products include free DNA, RNA products and nucleosomes, all of which can act as damage signals and trigger the inflammatory response. Elevated free DNA levels were observed in patients with SAP and sepsis on admission, however patients with milder disease had levels comparable to healthy controls (19). Plasma DNA levels were directly correlated with worsening severity of the CT score (radiological score of pancreatic injury). Micro RNAs (miRNAs) levels have been shown to increase in AP, significantly more so in SAP and with strong correlation with clinical scores of pancreatitis severity (41). Cold-inducible RNA-binding protein (CIRP) is an intranuclear protein that is activated by cellular stress and induces a proinflammatory response through a damage-signalling mechanism. In a prospective observational study of patients with SAP, MAP and healthy controls, admission serum CIRP levels were elevated in SAP patients, with these levels associated with increased risk of organ dysfunction, local pancreatic complications and mortality (14). Conversely, CIRP levels in MAP patients were comparable to those of healthy controls. A similar trend is observed with plasma nucleosome levels, with higher admission nucleosome levels in more severe disease, and therefore potential utility for nucleosomes as biomarkers of severity and prediction of organ dysfunction (217).

Heat shock proteins are intracellular proteins also activated in response to cellular stress, however these have protective effects on cells by inhibiting inflammatory mediators such as nuclear factor kappa-beta (NF-KB). In a small study, converse to the other DAMPs, Heat shock protein 70 (Hsp70) levels were lower on admission in SAP compared to MAP and MSAP, with sustained reduction in Hsp70 levels in the SAP cohort over the 7-day study period (10). Hsp70 levels were similarly depressed in non-survivors compared with survivors. A similar trend was observed for heat shock factor 1 (HSF1) levels in a case-control study, where SAP admission HSF1 levels were reduced compared to MAP and healthy controls (84). The elevated heat shock molecule levels in milder disease is postulated to exert a cytoprotective effect and inhibit the dysregulated inflammatory response, whereas the early disease progression in SAP may in part reflect failure of the heat shock systems.

### Pattern recognition receptors

PRRs expressed by immune cells serve as the receptors for the above damage signals. These receptors include toll-like receptors (TLRs), receptors for advanced glycosylation end products (RAGEs), nucleotide oligomerisation domain-like receptors (NLRs) and the absent-in melanoma-2 (AIM2) protein. Interaction between DAMPs and these receptors activates inflammatory responses and cytokine release, which subsequently triggers leukocyte recruitment to the site of tissue injury.

Higher TLR4 expression on peripheral blood mononuclear cells (PBMCs) in patients with AP has been reported, as compared to healthy controls (22). There were no differences in TLR4 expression between MAP and SAP, however these findings may have been confounded by the use of empiric glucocorticoids in the SAP cohort. These findings are supported by studies of mRNA derived from PBMCs that found high levels of TLR4 and TLR2 transcripts in AP as compared to healthy controls (49, 181). In summary, all three studies suggest higher TLR4 activity in AP compared to controls, however they do not determine whether TLR4 expression differs between mild and severe disease.

RAGEs act as receptors for HMGB-1, augmenting its inflammatory action. Interestingly, in a prospective case control study, plasma soluble RAGE (sRAGE) levels in SAP were significantly lower than healthy controls (19). In the same study, sRAGE levels in MAP patients and those with severe sepsis were significantly higher than the SAP cohort and were elevated compared to the healthy controls. In patients with severe sepsis, there was a direct correlation between HMGB-1 and sRAGE levels, whereas the relationship was inverse in the AP patients. The mechanism for this relationship is unclear and requires further delineation.

On activation by damaged molecules, AIM2 forms an intracellular inflammasome. Inflammasomes are cytosolic complexes that regulate proteolytic caspase-1 and the release of pro-inflammatory cytokines. On admission, patients with AP had higher levels of AIM2 compared to controls, with augmented expression in more severe disease (135). ROC AUC for AIM2 predicting SAP was high (0.945) suggesting it may be a useful admission biomarker for severity. NLRP3 is another inflammasome complex that that likely contributes to AP disease pathogenesis, contributing to both pro and anti-inflammatory cytokine pathways processes (218). The NLRP3 inflammasome has been extensively studied in animal models, but investigations in human AP are comparatively scarce. In one observational study, serum ASC levels, a molecule closely linked to the activity of the NLRP3 inflammasome and a surrogate marker of its activity, were measured in healthy controls and patients of varying degrees of AP severity (219). ASC levels were found to correlate with disease severity and IL-18 levels (see below), implicating NLRP3 in the pathogenesis of human AP, and warranting further investigation of this protein complex in human studies.

### Granulocyte activation

Increased expression of markers of leukocyte adhesion and activation, hallmarks of inflammation, have been reported in AP. With regards to the granulocytic response, a peripheral blood neutrophilia and monocytosis predominates in the acute phase, with higher counts observed in more severe disease (142). Adhesion molecules facilitate cellular migration from the blood vessels into the tissue to exert their inflammatory effects. A number of adhesion molecules have been identified as being involved early in the disease course of AP, including intercellular adhesion molecule-1 (ICAM-1 or CD54), vascular cell adhesion molecule-1 (VCAM-1), E-selectin, Integrin alpha L (CD11a) and Integrin alpha M (CD11b). These molecules are expressed both on peripheral blood monocytes (142), as well as by mononuclear cells isolated from acute pancreatic tissue (136). Expression of these molecules is increased in correlation with disease severity, indicating increased leukocyte activation in more severe AP, which likely perpetuates tissue injury and further drives the inflammatory response (12, 27, 136, 142, 191). The early elevation of these molecules in AP suggests their potential utility as biomarkers of severity.

### Fatty acid-induced lipotoxicity

AP is associated with high levels of circulating free fatty acids (FFAs), which is likely precipitated by elevated lipolytic enzymes at both the local pancreatic and systemic level (220). In a case control study comparing FFA levels in patients with and without necrotizing pancreatitis, FFA levels were elevated in both groups on admission, but normalized by day 7 in the group without necrotizing pancreatitis (221). In the necrotising pancreatitis cohort, FFA levels remained elevated, possibly indicating ongoing pancreatic and extrapancreatic fat necrosis. There is evidence that FFAs, including unsaturated fatty acids (UFAs) and non-esterified fatty acids (NEFAs) contribute to the inflammatory response and tissue injury by triggering pro-inflammatory cytokine activity as well as direct deleterious effects on mitochondrial function (222, 223). Investigating the pathophysiology of fatty acid lipotoxicity in humans patients with AP is comparatively limited and warrants further investigation.

## Cytokine profile

The cytokine profile in AP was investigated in 136 of the included studies. Establishing a temporal cytokine profile in AP has proven difficult, largely due to heterogeneity in the timing of the studies in regard to symptom onset. Of the 136 studies, only 40 (29%) measured the cytokine profile to day 7 of disease, 27 (20%) to day 14, and 11 (8%) beyond day 14.

DAMP recognition by TLRs on leukocytes triggers a cascade of cytokines, which significantly amplify the inflammatory response, help activate the adaptive immune system, and thereby, contribute to ongoing tissue injury. The literature consistently describes a profound cytokine release in AP, augmented in SAP compared to MAP, which likely accounts for the degree of illness and organ failure observed in patients with more severe disease. In parallel to the pro-inflammatory response is a concomitant release of anti-inflammatory cytokines, which promotes immunosuppression and contribute to secondary complications (such as infection) in patients with more severe disease.

In the studies reviewed, the cytokines elevated in AP compared to healthy controls included TNFα (99, 110), IFNγ (99, 109), IL-1 receptor antagonist (IL-1Ra) (76, 78), IL-2 (109, 150), IL-6 (10, 49, 83, 93), IL-8 (10, 87), IL-10 (70, 99) and IL-18 (107, 112), all of which are also consistently significantly differ between mild and severe disease.

TNFα and IL-6 are elevated early in the disease course of SAP, with the degree of elevation correlated to disease severity (10, 73, 75, 79, 82, 83, 87, 94, 99, 101). Both cytokines are also significantly higher in non survivors (13, 16, 73, 99). In the studies that reported timing of peak TNFα levels in SAP patients, the majority reported that TNFα peaked within the first 24-48 hours of study inclusion (22, 48, 51, 57, 64, 66, 74, 80, 115, 143, 171, 186, 203, 205, 208), although peak levels beyond this timeframe have also been reported (87, 102, 174, 212), which may reflect varying study timeframes and definitions of onset. Similarly for IL-6 in SAP patients, nearly all studies that temporally measured levels reported a peak within the first 24 hours of study inclusion (22, 44, 45, 55, 56, 60, 61, 64, 67, 68, 74, 75, 78, 80, 87, 91, 95, 111, 115, 143, 171, 174, 179, 183, 186, 190, 191, 195, 196, 203, 205, 208) although one outlier study did report a peak at day 6 (93). Some of the proposed effects in the acute inflammatory process include neutrophil chemoattraction, activation of NF-KB pathways to augment inflammatory signalling, stimulation of hepatic synthesis of acute phase proteins, and promotion of other pro- and anti-inflammatory cytokine release. Given their elevation early in the disease course and correlation with poor outcomes, both have been proposed as potential early biomarkers of disease severity. TNFα activity has also been assessed by measuring its soluble receptor subtypes 1 and 2, both of which are higher in more severe disease reflecting higher biological activity of TNFα (51, 57, 82). A similar pattern is observed for IL-8, which typically peaks within the first 48 to 72 hours of admission for patients with SAP, and exerts its effects *via* interactions with CXC Receptor 1 on neutrophils to cause cellular activation and tissue injury (10, 73, 79, 82, 83, 87, 101).

Interferon-gamma (IFNγ) is also a postulated significant mediator, given its potential to activate cells of both the innate and adaptive systems. IFNγ levels are reportedly higher at baseline in SAP compared to MAP, in non-survivors versus survivors, and in all AP compared to controls, which is consistent with a heightened inflammatory response (99, 109, 150). Subsequent variability in IFNγ levels have been observed beyond day 7, possibly due to the fluctuating activity and secretion by macrophages and lymphocytes at divergent time points (109). IL-18 has also been studied in AP, where it may act synergistically with IFNγ to activate Th1 lymphocyte subsets (112). Elevated circulating IL-18 have been reported at admission and into the second week of disease, and are pronounced in severe disease associated with necrosis, multi-organ failure, and in non-survivors (83, 97, 107, 112, 114, 224).

IL-2 is also implicated in the pathogenesis of AP, specifically in relation to T cell activation. Most studies measured IL-2 receptor subtypes as a marker of IL-2 activity. Higher levels of soluble IL-2 receptor (sIL-2R) are associated with more severe AP (69, 73, 76, 109), and IL-2 receptor antagonist (IL-2Ra or CD25) is significantly higher in SAP compared to MAP (83). However these results are in contrast to another study which reported a reduction in peripheral blood CD25 mRNA expression in SAP patients on admission compared to MAP (49).

A number of other cytokines have been postulated to contribute to the acute inflammatory response in AP, however results are more variable, and they have been less extensively studied. These include IL-4, IL-12, IL-13, IL-17 and IL-23. As the latter three cytokines largely define the Th17 lymphocyte subset (63, 178, 182), it raises the question of what role these pro-inflammatory cells might play in SAP, particularly given their suggested role in mucosal immunity. Further work is required to determine their contribution to SAP pathology.

In conjunction with pro-inflammatory cytokine release is an observed elevation in the plasma concentrations of the regulatory cytokine IL-10 in AP, which was higher in SAP than MAP (58, 73, 75, 99, 101, 110, 150). IL-10, while traditionally regarded as an anti-inflammatory cytokine, likely has pleiotropic effects in AP. Its pro-inflammatory effects may include activation of natural killer (NK) cells, enhanced B cell function and stimulation of cytotoxic molecule expression, which may contribute to the inflammatory response and tissue injury (99). However, its more predominant effects appear to be a counter-regulatory response that dampens inflammatory pathways, which may account for the greater degree of elevation seen in SAP compared to MAP. In SAP, its postulated downstream effects include reduced MHC-II expression (particularly HLA-DR) and down-regulation of NF-KB signalling. High IL-10 levels are correlated with a significantly higher rate of complications in patients with SAP, including higher mortality, higher risk of infectious complications (particularly infected pancreatic necrosis) and the development of sepsis (16, 58, 73, 75, 76, 79, 99, 101, 110, 150). A case-control study investigated cytokine production in leukocytes in culture from MAP and SAP patients in order to assess functional reserve and anergy (70). There was increased basal cytokine levels in SAP compared to MAP, including IL-10. Interestingly, with cellular stimulation, cells isolated from MAP patients produced additional IL-10 in conjunction with other cytokines, while in SAP there was only a rise in pro-inflammatory cytokines. This data suggests that IL-10 release is more regulated in patients with MAP and induces a protective effect, while in SAP there is perhaps excessive unregulated production of IL-10 in response to the hyperinflammatory response, which confers detrimental effects to the individual.

The antagonist for the IL-1 receptor (IL-1Ra) also contributes to the pattern of immunosuppression by binding the IL-1 receptor and thereby inhibiting the pro-inflammatory actions of IL-1. Reflecting its regulation by pro-inflammatory IL-6 and IL-1β, significantly higher levels of IL-1Ra are observed in patients with SAP compared to MAP, and these higher levels are associated with higher mortality, the development of organ dysfunction and infectious complications (73, 110, 166).

### Limitations and future directions of cytokine assessment in AP

Definitively determining the cytokine pathways upregulated in AP that contribute to the pathogenesis is of significant value in the current era of monoclonal antibodies and other targeted therapies. For example, many of the implicated cytokines are currently targeted in other disease states, including anti-TNFα and IL-1 (i.e. *via* infliximab and anakinra, respectively) in inflammatory arthritis and autoinflammatory disorders, IFNγ (via JAK inhibitor tofacitinib) in rheumatoid arthritis and inflammatory bowel disease as well as anti-IL-6 antibody tocilizumab in rheumatoid arthritis, juvenile idiopathic arthritis and severe COVID-19 (225). While the cytokine profile in AP has been extensively reported, there are several limitations in evaluating the data. Of the 137 studies that were selected for inclusion, 103 (75%) employed enzyme immunoassay techniques for cytokine measurement, however the specific assay type and/or manufacturer was not often reported, which can lead to inter-assay variability. Interpreting cytokine levels and trends is also made difficult by the inconsistency of timing of patient recruitment to the studies. However, further advancements in these areas have the potential to rationally advance anti-cytokine therapies into clinical trials in AP.

## Cytokine gene polymorphisms

As part of prediction of disease onset and severity, there is great interest in understanding whether genetic predispositions may contribute to the pathogenesis of AP. Nineteen studies investigated for genetic polymorphisms that may be implicated in the development of SAP. The majority of these (79%) were case-control studies. The median number of participants was 361, and 18 of the studies included a healthy control group. All studies compared mild and severe pancreatitis. Polymorphisms for genes encoding for cytokines, heat shock proteins and pattern recognition receptors have been investigated. Several polymorphisms were reported to be associated with an increased predisposition to the development of acute pancreatitis (all levels of severity) including IL-1Ra, IL-8, Il-23, CD14 and MMF (118, 120, 123, 124, 129, 131). However, while a number of polymorphisms were investigated to assess their correlation with more severe disease (polymorphisms in TLR3, TLR6, IL-1Ra, CD14, TNFa, MCP-1, receptor-interacting protein kinases (RIPK), myelin basic protein (MBP) and HSP70), statistical significance was not achieved in any of these studies (41, 117, 121, 124, 125, 127, 129, 131). Based on these findings, it remains unclear whether there are any clear genetic predispositions to the development of SAP. Given the number of polymorphisms identified in pro-inflammatory pathways, larger scale prospective genomic studies of AP in conjunction with metabolomic evaluation would be of great value.

## Lymphocyte profile

Twenty-nine studies investigated changes in the lymphocyte profile in patients with AP. As with cytokine studies, a notable limitation of these studies was the limited timeframe assessed; 14 studies (48%) did not assess the lymphocyte profile beyond one week, and 5 (17%) did not specify time points. Only 3 studies (10%) assessed the temporal profile up to day 30 of admission.

As is observed in other hyperinflammatory disorders, such as sepsis, generalised lymphopaenia typically occurs early in AP (often on admission), in both mild and severe disease (150, 161), with a greater degree of lymphopaenia seen in more severe pancreatitis (147, 161). Lymphopaenia is associated with higher risk of secondary infectious complications (156, 226). Lymphopaenia in AP is often associated with an elevated neutrophil count, and a high neutrophil to lymphocyte ratio (NLR) at admission can be a useful predictor of poor outcomes (147, 153, 226), similar to the sepsis syndrome (227). Regarding more detailed lymphocyte profiling, the majority of studies have explored T lymphocytes, which appear to be more affected in comparison to B cells (150). T cell subset analysis indicates multiple lines are depressed in AP including Cytotoxic CD8^+^ T cells, NK cells and CD4^+^ T cell counts (109, 142, 161). While both CD8^+^ and CD4^+^ T cell counts are diminished, a number of studies suggest that CD4^+^ counts are more significantly affected in more severe disease, causing reduced CD4:CD8 ratios (109, 115, 150).

While early lymphopaenia is a recognised phenomenon of AP severity, one study observed a biphasic trend in T cell dynamics (150); T cell counts were significantly reduced on admission but recovered to within normal range by day 10. T cell counts were again low at day 30, but this reduction was particularly marked in the SAP cohort. In the same study, B cell counts, while reduced compared to the control cohort, were essentially the same in both the MAP and SAP patients until beyond day 7, when the SAP B cell counts became significantly reduced compared to MAP. Innate cellular immunity may be further reduced by heightened activity of CD4^+^CD25^+^CD127^low/neg^ regulatory T cells (T_reg_) in more severe disease with associated poorer outcomes (144).

In keeping with an acute inflammatory response, elevated expression of lymphocyte activation markers is present in AP. CD69 is a surface marker of T cell activation and has significantly elevated expression in both mild and severe AP, both in the peripheral blood and at the pancreatic tissue level (136), as well as elevated CD69 mRNA expression in PBMCs of patients with AP (49).

## Immunodeficiency

In parallel to observed, marked inflammatory response, are regulatory immunosuppressive processes, the activity of which are more pronounced in patients with severe disease.

### HLA-DR expression in AP

As an MHC-II molecule, human leukocyte antigen-DR (HLA-DR) is a surface marker of cellular activation and plays a key role in T cell antigen presentation and regulation of pro-inflammatory cytokine release. Reduced HLA-DR expression is a reliable biomarker of immunodeficiency and is associated with a higher risk of infection (228). In patients with mild and moderate AP, there is an observed reduction in HLA-DR expression within the first few days of disease onset, however this returns to near-normal within the first week (75). In contrast, patients with SAP – particularly those with multi-organ failure – downregulated HLA-DR expression persists beyond the first week of illness and is associated with increased rates of secondary infectious complications (40, 42). In a study comparing HLA-DR expression in AP patients with and without infectious complications, the cohort that developed secondary infections exhibited significantly lower HLA-DR expression (28). A similar inverse correlation has been observed between HLA-DR expression and mortality (16). Given the association between HLA-DR expression and disease severity and complications, it has also been proposed as an early biomarker of disease severity.

### Co-inhibitory molecule activity in AP

Further evidence of immunosuppression in AP is presented by the heightened expression of the immune checkpoint molecules programmed cell death 1 (PD1) and programmed cell death ligand 1 (PD-L1). PD1/PD-L1 is a well-known immune inhibitory pathway involved in prohibiting T cell activation and proliferation as well as the induction of T cell apoptosis. Heightened PD1/PD-L1 expression can be a means of immune system evasion in metastatic malignancy, but is also a marker of T cell exhaustion, such as in septic shock where there is an associated higher risk of nosocomial infectious complications (229). Recent data implicates heightened activity of the PD1/PD-L1 system in severe pancreatitis, with greater expression in patients who develop secondary infectious complications (28). Similar activity has been observed with the soluble isoform of PD-L1 (sPD-L1), demonstrating higher sPD-L1 levels in more severe AP and in those with infectious complications (11). Immune checkpoint inhibitors targeting the PD-1 are currently in clinical trials in sepsis, to determine their efficacy in reversing T cell anergy and exhaustion (230) and findings here may have some relevance to synonymous phenomena in AP.

### Leukocyte anergy

In addition to quantitative deficiencies, there is increasing evidence to suggest functional impairment of both lymphocyte and monocyte populations is present in severe disease. A state of cellular anergy in SAP would certainly increase the risk of subsequent infectious complications. One study performed *in vitro* assessments of lymphocyte NF-KB signalling in response to incubation with microbial pathogens, comparing the lymphocytes of patients with SAP (with HLA-DR expression <80%) with those of healthy volunteers (146). T and B cells from the SAP cohort had significantly reduced responses, suggesting a degree of functional exhaustion. Cells were subsequently treated with TNFα and NF-KB activity measured; the lymphocytes from SAP patients had significantly diminished responses compared to controls, indicating reduced number of TNFα-responding cells. A similar study assessed *in vitro* monocyte phagocytosis in peripheral blood on admission in mild, moderate and severe cohorts of AP, and compared this to healthy controls. In all cohorts, phagocytic activity was increased, with activity positively correlating with disease severity (153). However, on subgroup analysis, patients who developed infectious complications had significantly reduced monocyte phagocytic activity. Phagocytic activity was then further stimulated by incubating lymphocytes with protein kinase C activator (PMA – a protein that enhances macrophage oxidative metabolism and phagocytic function). Monocytes from the mild, moderate and severe (without infectious complications) all had significantly higher oxidative activity, whereas monocytes from patients who later developed infectious complications had no response to PMA, indicating cellular metabolic dysfunction.

## Failure of the intestinal barrier in acute pancreatitis

The intestinal mucosa plays a key role as a barrier preventing translocation of enteric microflora from the intestinal lumen into the bloodstream. Twenty-five studies that reported changes in intestinal permeability or assessed for markers of bacterial translocation were included. While in general critical illness intestinal failure occurs in the setting of systemic hypoperfusion and organ dysfunction; however in AP, gut barrier failure occurs early (even as early as at the point of hospital admission) (165), and has been observed prior to the onset of multi-organ failure (162). This suggests that in addition to intestinal hypoperfusion secondary circulatory shock (167), there may also be local mechanisms and mediators arising from the inflamed pancreas that directly contribute to intestinal ischaemia and barrier dysfunction. Increased intestinal permeability has been consistently observed in both mild and severe disease, although to a greater extent in the SAP cohort (143, 165–167).

Failure of the gut barrier facilitates translocation of enteric organisms, as well as bacterial endotoxin and antigens into the portal circulation (143). Several studies assessed plasma endotoxin levels (a component of the Gram-negative bacterial cell wall) as a marker of translocation, with higher endotoxin levels associated with higher gut permeability and more severe disease (102, 162, 163, 165). Endotoxinaemia has also been assessed by measuring endotoxin core IgM and IgG production, however this appears to be a less consistent method with studies reporting both significant elevation and reduction in EndoCAb IgM levels in mild and severe cohorts (140, 163, 166, 167, 169, 198). Endotoxin has been posited to enhance the inflammatory response *via* cytokine pathways as well as *via* direct stimulation of B cells (163, 167). Translocated organisms are likely to interact with pathogen-associated molecular patterns (PAMPs), triggering further inflammatory responses with leukocyte activation and cytokine release. It has been hypothesised that translocated intestinal bacteria are the source of infected necrosis, largely due to culprit organisms typically being Gram-negative enteric species.

While there is evidence supporting gut dysfunction and the presence of endotoxaemia in AP, there is little understanding in terms of immunopathological mechanisms and how these factors interplay. One study reported that the degree of endotoxinaemia did not correlate with the inflammatory cytokinaemia (59), while another observed that TNFα levels correlated with the degree of intestinal permeability (165). While this increased gut permeability almost certainly contributes to intra-abdominal infection, there is currently no strong evidence to suggest a mortality benefit from empiric antimicrobial therapy in AP (of any severity) (231). Overall, the interaction between increased gut permeability and the relationship to AP and sequelae of severe disease requires ongoing investigation.

## Intervention studies in acute pancreatitis

While there is currently an ongoing trial of anti-TNFα antibody (infliximab) in AP (RAPID-I) (232), there are relatively few completed trials of immunomodulation in AP in humans. We included 45 studies in which an intervention was trialled in AP, where either the intervention or an outcome had an immunological basis. Most studies included in this review assessed immunological outcomes such as trends in cytokine levels, cellular markers or immunoglobulin levels. The trialled interventions can be broadly categorised into drug therapies (n=18), intravenous (IV) fluid studies (n=2), nutritional studies (n=13) and extracorporeal therapies (n=12).

### Pharmacological interventions

Several pharmacological interventions have been trialled in AP with limited to no efficacy in reducing mortality despite improving biochemical, clinical and immunological markers.

Given the profound inflammatory response associated with SAP, glucocorticoids have been trialled albeit with no high quality RCTs testing their efficacy in this disease. We identified three studies that trialled glucocorticoids; one retrospective propensity-matched cohort study, one case control study and one case series (233–235). In the propensity matched cohort study, it is unclear what the indication for glucocorticoid therapy was, and patients were treated with varying formulations at differing doses and durations. In the glucocorticoid arm, compared to the propensity matched arm, there were improvements in mortality, ICU length of stay (LOS) and treatment costs. In the case control study, patients were treated with with 1mg/kg dexamethasone three times daily compared to standard care; the steroid arm had improved rates of ARDS and LOS but no improvement in mortality. This study was confounded by the use of Chinese herbal preparations in both groups, the effect of which on AP is unclear. Finally, the case series administered variable doses of dexamethasone and Dextran colloid solution with improvements in symptoms however in the absence of a control group, it is difficult to draw definitive conclusions. Across all studies, the variable definitions of severity, treatment protocols and patient heterogeneity, as well as the lack of immunological outcomes, makes the findings from these studies largely exploratory.

Several studies have trialled pancreatic enzyme inhibitors including somatostatin, ulinastatin, octreotide and gabexate. These agents were proposed to reduce enzyme activation and minimise the initial tissue injury, which may therefore mitigate the subsequent inflammatory and immune response. Findings were variable with some evidence for improved immunological markers including increasing the CD4:CD8 ratio and reduced pro-inflammatory cytokine expression (TNFα, IL-6, IL-8), but importantly a reduction in mortality or length of stay was not demonstrated (188, 200, 203, 207).

Similarly, pentoxifylline, a non-selective phosphodiesterase inhibitor, has been trialled in AP with variable effect on improving serum cytokine levels but no clear evidence of mortality benefit (175). The trials in this field are limited by low participant numbers and lack of clarity regarding baseline physiological status of participants. Other confounding factors that varied across intervention studies included what was considered ‘standard care’, including the empirical administration of antibiotics and corticosteroids (188, 192, 208).

Lornoxicam, a non-selective cyclooxygenase inhibitor, was tested in patients with SAP compared to standard care in a randomised, non-blinded clinical trial (181). Patients were randomised to standard care (n=246) or lornoxicam plus standard care (n=88). The trial demonstrated a temporally significant reduction in TLR2 mRNA expression in the lornoxicam group. The treatment arm also demonstrated significantly reduced mortality and fewer gastrointestinal complications. These results are potentially of interest, however must be interpreted with caution as the study was underpowered for mortality (which also was not a reported primary or secondary outcome), it is unclear whether true randomisation occurred, participant baseline clinical and biochemical data were not reported (therefore differences in outcomes may be contributed to by cohort heterogeneity), and there was a significant difference in the cohort sizes which may confound the magnitude of effect.

Randomised trials have been conducted to assess the effects of lexipafant, antagonist to platelet activating factor, as well as activated protein C (APC) in improving mortality and immunological outcomes in AP. No significant improvements in immunological outcomes or overall mortality were observed for either therapy (189, 191).

A potential limitation of these drug therapies is that they target the early disease process; that is, reducing pancreatic secretions and initial inflammation rather than suppressing the subsequent immune responses. Given the systemic immune dysregulation has likely already onset by the time patients present to hospital, this probably explains why these pancreas-specific treatments have no benefits from an immunological outcome’s perspective.

### Intravenous fluid

There have been four RCTs of lactate-containing balanced crystalloid solutions with 0.9% sodium chloride (0.9% saline) in patients with AP (236–239). The outcomes of these studies include frequency and duration of SIRS, clinical outcomes (e.g. mortality and LOS), and inflammatory markers (e.g.CRP). Patients administered the lactate-containing crystalloid had trends towards reduced duration of SIRS, reduction in CRP and some improved clinical outcomes such as ICU LOS but no impact on mortality. It is hypothesised that lactate-based solutions may confer anti-inflammatory effects by raising pH in the pancreatic tissue. Furthermore, these studies administered high volume fluid resuscitation based on goal-directed therapy protocols. In other critical care cohorts such as sepsis, goal-directed fluid therapy has since been robustly shown to not improve outcomes (240) and high volume fluid therapies are associated with an increased trend to harm across numerous critical care cohorts (including the recent WATERFALL trial in AP (241)). Finally, these studies enrolled patients with all severities of AP, which further limits the ability to draw conclusions on the potential benefits of different crystalloids in this disease. While these study findings are of interest, the studies were underpowered and therefore these findings should be considered hypothesis-generating.

Two studies have examined the effect of IV fluid on inflammation in SAP by comparing 0.9% saline solution to various formulations of hydroxyethyl starch (HES). Compared to NS, patients with AP who received a starch-based solution had some temporal improvements in cytokine levels (174, 212). It is hypothesized that HES may play some role in attenuating the inflammatory response, in particular capillary leakage *via* NK-κB activation, and neutrophil adhesion and migration. However, starch-based solutions have been demonstrated to increase the risk of acute kidney injury in critical illness and therefore cannot be readily considered as a potential therapy (242).

### Nutrition

Thirteen studies examined the effect of various nutritional approaches in SAP and their effect on the immune and/or inflammatory response. Overall, little insight into the immunopathology could be gained as many studies examined few time points, did not report specific cytokine levels, and primarily focused on non-immunological outcome measures such as organ failure and length of stay.

With respect to cytokines, total parenteral nutrition (TPN) adjuncts demonstrated some effect in reducing the inflammatory response compared to TPN alone e.g. fish oil was associated with reduced IL-8 and ICAM-1 (243), and increased IL-10 (204), however no difference was seen in sTNFR levels (194) or IL-6 (206). In comparing TPN and enteral nutrition (EN), EN was frequently demonstrated to be superior in respect to cytokine response - with reduced pro-inflammatory IL-6 (173, 202, 205), IL-1b (173), and TNFα (173, 205), and increased anti-inflammatory IL-11 and IL-10 (202, 205).

In regards to the cell-mediated response, TPN adjuncts such as glutamine increased lymphocyte count (194), and fish oil increased HLA-DR expression (204). HLA-DR expression also significantly increased with early EN compared to delayed EN (199).

In terms of gut permeability, multiple studies have demonstrated EN significantly reduces gut permeability compared to TPN *via* various measures including EndoCAb IgG and IgM antibodies (185, 209), endotoxin, and lactulose-mannitol ratios (205, 211). Supplementation of EN with glutamine was demonstrated to improve gut permeability and significantly reduce IL-6 (172) and endotoxaemia (198), however this did not translate into an improvement in infectious complications, morbidity or mortality. There is no evidence to suggest any immunological benefit from probiotic use in AP (197), and their use is actually associated with increased mortality in patients with SAP (244).

Finally, in terms of clinical outcomes, compared to TPN, EN was associated with lower rate of complications end organ dysfunction, as well as improved outcomes (173, 199, 202, 209).

### Extracorporeal therapies

Eleven studies investigated the effect of extracorporeal therapies in modulating the inflammatory response in patients with AP, predominantly those with severe disease. The aim of these therapies is to sequester inflammatory mediators from the plasma *via* dialysis, haemofiltration, or adsorption using specialized membranes.

Overall, it appears that extracorporeal therapies may be effective in reducing the plasma levels of inflammatory cytokines. Reduced cytokine levels were observed as early as within 6 hours of commencement of continuous veno-venous haemofiltration (177, 178), and high volume haemofiltration was associated with greater cytokine reduction than continuous filtration (176). In other studies, application of extracorporeal therapies was associated with improved clinical outcomes including reductions in mortality, improved parameters of respiratory function and improved haemodynamic outcomes (170, 183, 184). Wang et al. (208) randomised patients to standard care versus either haemofiltration or laparoscopic peritoneal lavage or haemofiltration plus peritoneal lavage. All intervention arms had improved clinical outcomes and maximal reduction in pro-inflammatory cytokines, however the greatest improvements were observed in the group who underwent both haemofiltration and lavage. This suggests an additional benefit may be derived from addressing the intra-abdominal inflammatory processes *via* lavage (i.e. removal of necrotic tissue, inflammatory mediators, and translocated intestinal flora). These findings are however confined to a single study and the potential benefits of this intervention would need to be balanced against the risks of significantly invasive surgery in an already critically ill patient population.

While these findings are promising, they were not consistent across the studies. Furthermore, methodological issues including lack of true randomisation, the absence of control groups, heterogenous study populations, variable definitions of disease severity, and low participant numbers make these observations hypothesis-generating rather than truly demonstrating therapeutic effect which would likely require further randomized interventional trials. Extracorporeal therapies are not without risk and these must be considered against any potential benefit. Inconsistency in the reporting of particularly outcomes, including the frequent measurement of non-standardised clinical outcomes such as ‘relief of abdominal distension’, makes comparison of results between studies challenging.

## Current research gaps and future directions

SAP is a potentially lethal condition in both the hyperacute and subacute phase with limited high-level evidence to guide management (245). The findings of this scoping review centering human translational data provide a compelling argument for dysregulation at multiple levels of the immune and inflammatory response, particularly the phenomena of concomitant pro and anti-inflammatory processes. While it is evident that there are derangements at many levels, which of these are causative and which are para-immune phenomena requires further clarification. The critical pathogenic steps in the immunobiology of acute pancreatitis remain elusive and clearly still require further considered observational and interventional study.

Of interest is that patients may progress to severe disease irrespective of the underlying aetiology of AP, suggesting an underlying susceptibility to dysregulated immune responses to tissue injury. The challenge is to now improve our understanding of the chronology of these changes, and to look beyond just the short term, if we are to improve our understanding of the biology of this disease. While our review has demonstrated the extensive work that has been conducted on the cytokine patterns, cellular profiles and innate immune responses in AP, transcriptomic analysis has not yet been conducted in humans Employing newer techniques such as RNA sequencing and transcriptomics may enable us to understand the temporal profile of the disease and the immunological endotype at the cellular level.

The COVID-19 pandemic has been invaluable in demonstrating the benefits of early diagnosis and immunomodulation to improve outcomes in critically ill patients with an acute inflammatory illness. A collaborative international approach to COVID-19 has led to the uptake of diagnostic biomarkers and establishment of clear management guidelines, which has enabled the application of personalized medicine. A similar approach and framework could have innumerable benefits in AP.

There is increasing interest in immune modulation for the management of pancreatitis, especially at the severe end of the spectrum. A recently published RCT (published following our literature search) compared thymosin alpha (an enhancer of cell-based immunity) with placebo in patients with predicted SAP (246); the primary outcome was development of infected pancreatic necrosis and the secondary outcomes included HLA-DR expression. In this study, participants were eligible for inclusion if they had had abdominal symptoms for up to 7 days. No statistically significant differences were observed between the treatment and control arms for either primary or secondary outcome, although there was a suggestion of increased benefit in patients with more extensive pancreatic necrosis. Interestingly, a recent observational study (also published beyond our inclusion dates) studied patients with infected pancreatic necrosis (IPN) longitudinally up to 60 days post-discharge and compared them with healthy controls (247). Patients with IPN had evidence of significant HLA-DR downregulation at 60 days post-discharge from the index admission, and patients who were readmitted to hospital within the time period had significantly lower HLA-DR levels compared to those who were not readmitted.

Our findings in this review suggest that given the rapidity of onset of the acute inflammatory response in SAP, allowing recruitment up to 7 days in the thymosin alpha study may potentially miss a ‘golden window’ for early immunological intervention, which could explain why no differences were observed between the two groups. The prolonged period of immune convalescence observed in the IPN study highlights the need to evaluate patients beyond the hospital period to better understand the long-term immunological profile in this patient group. For this reason, it is even more essential that more in depth research in AP is conducted to a more aligned standard, to allow for more accurate characterization of the acute natural history of this disease at the cellular and molecular level. Given the heterogeneous nature of the patient population and variability in rate of disease progression, this may explain why trials of interventions are yet to show positive outcomes. For example, the findings of this review suggest that anti-protease therapies are unlikely to be beneficial beyond the initial onset of the disease, while anti-cytokine therapies or corticosteroids may only be beneficial in the subsequent hyperacute phase of the illness, and they could potentially be harmful once the compensatory anti-inflammatory responses have established (248). These challenges further the need for biomarker-led interventions in AP clinical trial design.

We await with interest the findings from the RAPID-I multi-centre RCT that is comparing infliximab with placebo in AP and will employ transcriptomics to hopefully shed further light on the biology of this illness (232).

This review highlights the extensive work to-date that has been conducted into the immunobiology of human AP. While the developments are exciting, there are clear inconsistencies across several domains that will need to be addressed if meaningful progress is to be made in this area. Heterogeneity in the individual study definitions of pancreatitis and its severity has significant downstream implications for comparison of results and prospective study design. At the participant level, it is imperative that studies develop clear cut-offs for symptom duration prior to inclusion, in order to establish a degree of standardization between studies, given the rapidity dynamic nature of the disease progression. At present, diagnostic criteria for AP disease severity is largely retrospective—that is, the Revised Atlanta 2012 Consensus criteria (2) requires organ dysfunction to be present for 48 hours in order to define severe disease. It may be advantageous then to further evolve diagnostic criteria for pancreatitis studies to reflect molecular and immunological measures (e.g. IL-6 levels or HLA-DR expression). The benefits would include earlier, more accurate stratification of patients, which would facilitate earlier intervention, as well as developing more robust clinical trials by refining study cohorts. At the investigation level, there also needs to be standardization of assays to enable comparison of findings between studies. Finally, regarding outcome measurement, we propose the development of a set of core outcomes that should be reported in trials of severe pancreatitis inflammation and immunology.

## Conclusion

The pathobiology of AP is characterised by immune dysregulation in response to tissue injury, with the severity of illness correlated with the extent of immune response. The literature pertaining to human data implicates a number of pro-inflammatory pathways as well as features such as lymphopaenia and other regulatory elements that may indicate co-existing immunodeficiency. Inconsistencies in study design and our ability to prospectively assess severity have limited progression in our understanding of the immunopathology of this disease. Further well-designed, prospective studies that assess this disease at a detailed cellular, genomic and microbiome level are required if effective disease-modifying therapies are to be established.

## Author contributions

All authors contributed to the article and approved the submitted version. All authors were involved in study conception, methods and design. KV and HG performed the search, data collection, analysis and manuscript preparation. SS and CA performed data analysis, manuscript preparation and editing. AD contributed to manuscript preparation and editing.
