# Supplementary material for: Fire in the belly: A scoping review of the immunopathological mechanisms of acute pancreatitis
Source: Front Immunol. 2023 Jan 11;13:1077414. doi: 10.3389/fimmu.2022.1077414 (PMC9874226; doi:10.3389/fimmu.2022.1077414)
Supplement: Supplementary file 1 [file DataSheet_1.docx]

**Supplementary Data A: Search strategy with MeSH terms**

Acute disease AND human

AND

Acute pancreatitis OR Necrotising pancreatitis

AND

Histopathology OR immunopathology OR genome OR transcriptome OR proteome OR metabolome OR leukocytes OR lymphocytes OR T-cells OR B-cells OR cytokines OR chemokines OR interleukin OR interferon OR tumour-necrosis factor OR toll-like receptors OR intracellular messengers OR cytosolic calcium OR flow cytometry OR antibodies OR auto-antibodies OR immunomodulatory therapies
