# Supplementary material for: Fire in the belly: A scoping review of the immunopathological mechanisms of acute pancreatitis
Source: Front Immunol. 2023 Jan 11;13:1077414. doi: 10.3389/fimmu.2022.1077414 (PMC9874226; doi:10.3389/fimmu.2022.1077414)
Supplement: Supplementary file 2 [file Table_1.docx]

Supplementary Data B: Data Collection Form

**General information**

| **Study ID** |  |
| --- | --- |
| **Title** |  |
| **First author** |  |
| **Year published** |  |

**Study characteristics**

| **Classification of disease severity - Atlanta criteria specified?** | - Yes - No |
| --- | --- |
| **Methods** |  |
| Primary study focus | - Cytokine - Innate immunity - Leukocyte mediated - Genetic - Gut permeability - Intervention |
| Additional study domain(s) | - Cytokine - Innate immunity - Leukocyte mediated - Genetic - Gut permeability - Intervention |
| Types of samples collected | - Plasma - Whole blood - Tissue - Radiology - Other |
| Study design | - Randomised controlled trial - Pseudo-RCT - Cohort study (prospective or retrospective) - Case control study - Case series - Other |
| Number of centres/hospitals |  |
| Country |  |
| **Participants** |  |
| Pancreatitis severity | - Mild - Moderate - Severe - Not specified |
| Time since symptom onset (days) |  |
| Pancreatitis aetiology | - Biliary/gallstone - Alcohol - Post-ERCP - Drug-induced - Hypertriglyceridaemia - Other |
| Ethnic subset |  |
| Total number of participants |  |
| Control group present | - Yes - No |

**Cytokine studies**

| **Time points clearly specified** | - Yes - No |
| --- | --- |
| **Time points of cytokine measurement** | - Day 0 ☐ Day 8 - Day 1 ☐ Day 9 - Day 2 ☐ Day 10 - Day 3 ☐ Day 11 - Day 4 ☐ Day 12 - Day 5 ☐ Day 13 - Day 6 ☐ Day 14 - Day 7 ☐ Beyond day 14 |
| **Cytokines being measured**  (including their associated receptors and subtypes) | - TNFα ☐ IL-10 - IFNɣ ☐ IL-12 - IL-1 ☐ IL-17 - IL-2 ☐ IL-18 - IL-6 ☐ IL-23 - IL-8 ☐ Other (specify below) |
| **Other cytokines - specify** |  |
| **Cytokine time course** |  |
| Trend (P = peak /T = trough); Timing of peak vs. trough (# = day); SAP vs other AP ?significant (Y/N); SAP change from baseline ?significant (Y/N); SAP (peak) vs. control (peak) ?significant (Y/N); Units of measurement (eg. Pg/ml)**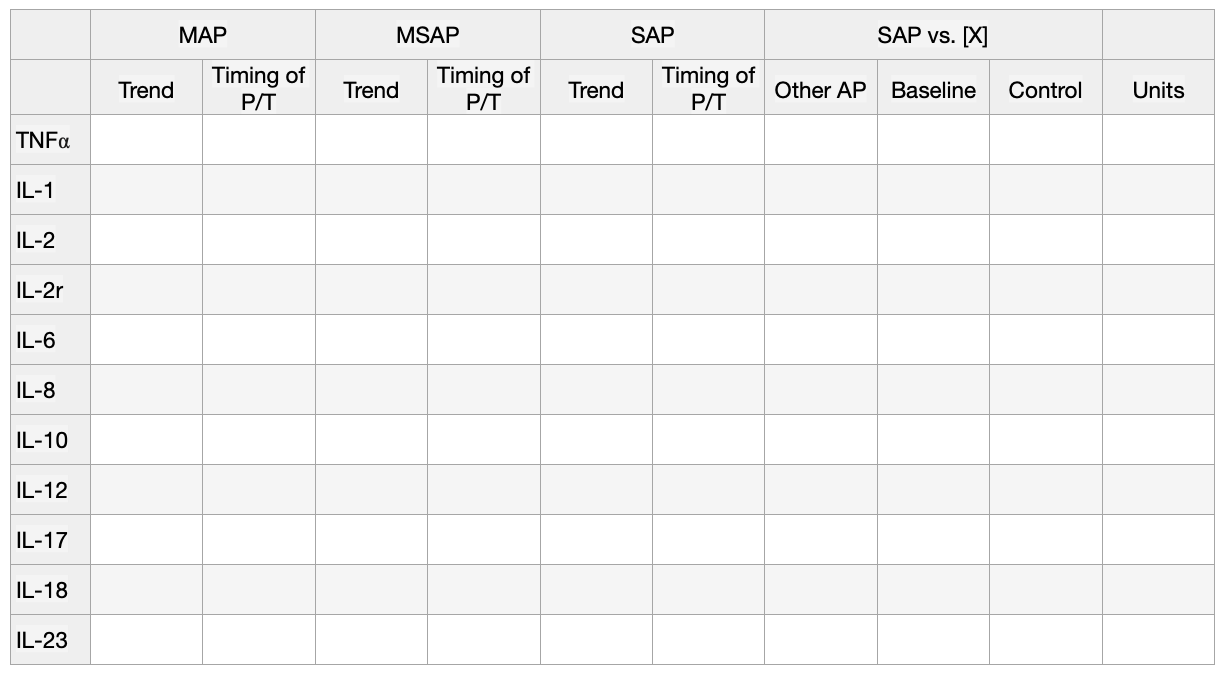** | |
| **Assay technique** | - Enzyme immunosorbent assay (ELISA, EIA) - Chemiluminescence - Not specified - Other |
| **Key findings summary** |  |

**Innate immunity studies**

| **Marker being assessed** | - HLA-DR - Toll-like receptor (TLR) - Inflammasome (specify next) - DAMP/PAMP (specify next) - Other intra/intercellular messenger (specify next) |
| --- | --- |
| **Inflammasome - specify** |  |
| **DAMP/PAMP - specify** |  |
| **Other messenger - specify** |  |
| **Assay used** |  |
| **Key findings summary** |  |

**Leukocyte studies**

| **Cell types being measured** | - CD4+ lymphocytes - CD8+ lymphocytes - Other lymphocytes (NK cells, others) - Peripheral blood mononuclear cells - Neutrophils - Other granulocytes (basophils/eosinophils) |
| --- | --- |
| **Cell lines**  Cell-line (lymphocytes, monocytes, NK, etc); Marker (ICAM1, CD#, etc); Key findings (1-3 dot points) | Cell type Marker(s) Key findings  Cell 1  Cell 2  Cell 3  Cell 4  Cell 5 |
| **Time points of measurement** | - Day 0 ☐ Day 8 - Day 1 ☐ Day 9 - Day 2 ☐ Day 10 - Day 3 ☐ Day 11 - Day 4 ☐ Day 12 - Day 5 ☐ Day 13 - Day 6 ☐ Day 14 - Day 7 ☐ Beyond day 14 |
| **Cellular pathway measured** | - Activator - Suppressor - Both |
| **Key findings summary** |  |
| **Long term (post-discharge) immunological follow up** | - Yes - specify - No |
| Outcome measure |  |
| Timing of follow up |  |

**Genetic studies**

| **Gene(s)** |  |
| --- | --- |
| **Key findings summary** |  |

**Gut permeability studies**

| **Method of measuring gut permeability** | - Assay - specify - Polyethylene glycol absorption / urinary excretion - Other |
| --- | --- |
| Specify |  |
| **Biomarkers measured** | - Endotoxin - EndoCAb IgG/IgM - Other |
| **Outcome measure(s)** |  |
| **Key findings summary** |  |

**Intervention studies**

| **Intervention type** | - Drug - Nutrition - IVF - Dialysis/filter - Other |
| --- | --- |
| **Specific intervention** |  |
| **Control group intervention** | - IV crystalloid - Antibiotics - Enteral nutrition - Parenteral nutrition - Analgesia - Other |
| **Outcome measures** | - Mortality - Length of stay - Clinical parameters (abdominal pain, vomiting, nausea, distension, other) - Routine inflammatory markers (CRP, ESR, WCC) - Research markers (cytokines, cell surface markers, innate immune markers, other) |
| **Specific immune measures** |  |
| Cytokines | - Yes - No |
| Cellular outcomes (lymphocytes, immunosuppression, Immunoglobulins) | - Yes - No |
| Innate immune markers | - Yes - No |
| Clinical parameters | - Yes - No |
| **Clear time points specified for outcome measures** | - Yes - No |
| **Key findings summary** |  |

**Histopathology findings**

| **Key findings summary** |  |
| --- | --- |
